# Integrative Multimodal Metabolomics to Early Predict Cognitive Decline Among Amyloid Positive Community-Dwelling Older Adults

**DOI:** 10.1093/gerona/glae077

**Published:** 2024-03-07

**Authors:** Marie Tremblay-Franco, Cécile Canlet, Audrey Carriere, Jean Nakhle, Anne Galinier, Jean-Charles Portais, Armelle Yart, Cédric Dray, Wan-Hsuan Lu, Justine Bertrand Michel, Sophie Guyonnet, Yves Rolland, Bruno Vellas, Julien Delrieu, Philippe de Souto Barreto, Luc Pénicaud, Louis Casteilla, Isabelle Ader, MAPT/DSA Group

**Affiliations:** Toxalim (Research Center in Food Toxicology), Toulouse University, INRAE, ENVT, INP-Purpan, UPS, Toulouse, France; Metatoul-AXIOM Platform, MetaboHUB, Toxalim, INRAE, Toulouse, France; Toxalim (Research Center in Food Toxicology), Toulouse University, INRAE, ENVT, INP-Purpan, UPS, Toulouse, France; Metatoul-AXIOM Platform, MetaboHUB, Toxalim, INRAE, Toulouse, France; Institut RESTORE, UMR 1301 INSERM, 5070 CNRS, Université Paul Sabatier, Toulouse, France; Institut RESTORE, UMR 1301 INSERM, 5070 CNRS, Université Paul Sabatier, Toulouse, France; Institut RESTORE, UMR 1301 INSERM, 5070 CNRS, Université Paul Sabatier, Toulouse, France; Institut Fédératif de Biologie, CHU Purpan, Toulouse, France; Institut RESTORE, UMR 1301 INSERM, 5070 CNRS, Université Paul Sabatier, Toulouse, France; MetaboHUB-MetaToul, National Infrastructure of Metabolomics and Fluxomics, Toulouse Biotechnology Institute, INSA de Toulouse INSA/CNRS 5504 - UMR INSA/INRA 792,Toulouse, France; Institut RESTORE, UMR 1301 INSERM, 5070 CNRS, Université Paul Sabatier, Toulouse, France; Institut RESTORE, UMR 1301 INSERM, 5070 CNRS, Université Paul Sabatier, Toulouse, France; Gérontopole of Toulouse, Institute of Aging, Toulouse University Hospital (CHU Toulouse), Toulouse, France; CERPOP UMR 1295, University of Toulouse III, INSERM, UPS, Toulouse, France; Lipidomic, MetaboHUB-MetaToul, National Infrastructure of Metabolomics and Fluxomics, Toulouse, France; I2MC, Université de Toulouse, Inserm, Université Toulouse III - Paul Sabatier (UPS), Toulouse, France (Biological Sciences Section); Gérontopole of Toulouse, Institute of Aging, Toulouse University Hospital (CHU Toulouse), Toulouse, France; CERPOP UMR 1295, University of Toulouse III, INSERM, UPS, Toulouse, France; Gérontopole of Toulouse, Institute of Aging, Toulouse University Hospital (CHU Toulouse), Toulouse, France; CERPOP UMR 1295, University of Toulouse III, INSERM, UPS, Toulouse, France; Gérontopole of Toulouse, Institute of Aging, Toulouse University Hospital (CHU Toulouse), Toulouse, France; CERPOP UMR 1295, University of Toulouse III, INSERM, UPS, Toulouse, France; Gérontopole of Toulouse, Institute of Aging, Toulouse University Hospital (CHU Toulouse), Toulouse, France; CERPOP UMR 1295, University of Toulouse III, INSERM, UPS, Toulouse, France; Gérontopole of Toulouse, Institute of Aging, Toulouse University Hospital (CHU Toulouse), Toulouse, France; CERPOP UMR 1295, University of Toulouse III, INSERM, UPS, Toulouse, France; Institut RESTORE, UMR 1301 INSERM, 5070 CNRS, Université Paul Sabatier, Toulouse, France; Institut RESTORE, UMR 1301 INSERM, 5070 CNRS, Université Paul Sabatier, Toulouse, France; Institut RESTORE, UMR 1301 INSERM, 5070 CNRS, Université Paul Sabatier, Toulouse, France

**Keywords:** Alzheimer’s disease, Bimodal metabolomics, Cognitive decline, Early prediction, Metabolite signature, Multiomics integrative method

## Abstract

Alzheimer’s disease is strongly linked to metabolic abnormalities. We aimed to distinguish amyloid-positive people who progressed to cognitive decline from those who remained cognitively intact. We performed untargeted metabolomics of blood samples from amyloid-positive individuals, before any sign of cognitive decline, to distinguish individuals who progressed to cognitive decline from those who remained cognitively intact. A plasma-derived metabolite signature was developed from Supercritical Fluid chromatography coupled with high-resolution mass spectrometry (SFC-HRMS) and nuclear magnetic resonance (NMR) metabolomics. The 2 metabolomics data sets were analyzed by Data Integration Analysis for Biomarker discovery using Latent approaches for Omics studies (DIABLO), to identify a minimum set of metabolites that could describe cognitive decline status. NMR or SFC-HRMS data alone cannot predict cognitive decline. However, among the 320 metabolites identified, a statistical method that integrated the 2 data sets enabled the identification of a minimal signature of 9 metabolites (3-hydroxybutyrate, citrate, succinate, acetone, methionine, glucose, serine, sphingomyelin d18:1/C26:0 and triglyceride C48:3) with a statistically significant ability to predict cognitive decline more than 3 years before decline. This metabolic fingerprint obtained during this exploratory study may help to predict amyloid-positive individuals who will develop cognitive decline. Due to the high prevalence of brain amyloid-positivity in older adults, identifying adults who will have cognitive decline will enable the development of personalized and early interventions.

Alzheimer’s disease (AD) affects over 35 million people worldwide and is anticipated to affect 115 million people by 2050 (1). To date, no treatment can reverse the clinical progression of the disease, especially in its later stages. Early detection is crucial from a clinical and societal point of view, potentially enabling the use of preventive strategies to fight against memory loss, cognitive decline, and functional impairment. Amyloid-beta (Aβ) peptide accumulation is thought to be an early trigger and marker of AD pathophysiology, and total amyloid load increases the risk of cognitive decline onset (2). However, only some individuals with amyloid accumulation experience cognitive decline. The identification of patients with an increased risk of cognitive decline is a major challenge to better determine those who might best benefit from innovative therapies (3) and will require additional metrics beyond amyloid load for better prediction.

AD is strongly linked to aging, which is accompanied by cognitive decline, memory loss, metabolic dysregulation, bioenergetic abnormalities, and inflammation. Studies have linked metabolomic profiles to aging (4), disease onset (5,6), and mortality (7), demonstrating that the human blood metabolome directly reflects physiological status. However, although perturbations in metabolism are widely recognized to be related to aging, few links have been established between systemic abnormalities in metabolism and cognitive decline (8). The healthy brain is the key organ that controls an individual’s homeostasis, and its energy metabolism is fueled exclusively by metabolites such as glucose and ketone bodies. Furthermore, the lipid rheostat is essential for proper brain function. Therefore, the global monitoring of both lipid and polar metabolites is essential to assess as early as possible the decline of brain health, which is usually manifested by cognitive decline. In this context, metabolomics has become a powerful phenotyping tool, in which measurements of metabolites at scale enable a molecular understanding of (patho)physiology and identification of biomarkers of metabolic deviations (9–11). Compared to clinical assessments based on single metabolites, metabolic signatures provide a direct input and readout of aging processes, which can reveal subtle key metabolic changes and ultimately stratify health trajectories (12). Several metabolomics studies using proton (^1^H) nuclear magnetic resonance (NMR) or mass spectrometry (MS) on blood samples have identified many altered metabolites in patients with dementia compared with healthy controls (13,14). Nevertheless, these studies were cross-sectional, capturing single time points. Longitudinal studies considering the development of cognitive decline that occurs over a long period of time could connect metabolic changes to clinical phenotypes.

In this exploratory study, we aimed to identify a panel of metabolic biomarkers that could be used to predict cognitive decline at the early stages of amyloid accumulation before clinical symptoms manifest. To fulfill this objective, amyloid-positive subjects from the MAPT study, categorized as cognitive decliners or non-decliners over up to a 4-year follow-up, were selected and plasma samples at inclusion were analyzed by determining the plasma-derived metabolite signature of each individual.

## Materials and Methods

### Clinical Assessment

#### MAPT study

Plasmas samples were obtained from the Multidomain Alzheimer Preventive Trial (MAPT, ClinicalTrials.gov [NCT00672685]), a randomized, multicenter, placebo-controlled trial conducted with community-dwelling older adults in France and Monaco. Participants were allocated into 4 groups, either receiving ω-3 polyunsaturated fatty acid (PUFA) supplementation, a multidomain intervention (based on cognitive training, nutritional counseling, and physical activity advice), both, or placebo. The intervention lasted for 3 years and was followed by an additional 2-year observational phase. Recruitment of participants started in May 2008 and ended in February 2011. Follow-up ended in April 2016 (15). A detailed description of the MAPT study can be found elsewhere (15). In summary, eligibility criteria comprised: age 70 years or older; not presenting major neurocognitive disorders, Mini-Mental State Examination [MMSE] score ≥24; presenting at least 1 of the following: spontaneous memory concern, inability to perform 1 instrumental activity of daily living (IADL), or slow usual-pace walking speed (<0.8 minutes/seconds). The present study followed the Strengthening the Reporting of Observational Studies in Epidemiology (STROBE) guideline (16).

#### Ethical aspects

The study was approved by the French Ethics Committee located in Toulouse (CPP SOOM II) and authorized by the French Health Authority. Written consent was obtained from all participants. The protocol is registered on the clinical trials database (www.clinicaltrials.gov-NCT00672685).

#### Definition of subgroups analysis

Twenty-four sex-matched participants, half decliners, and half non-decliners provided blood samples for this exploratory study, but only 20 participants had usable metabolomics data. Decliners/non-decliners were classified according to overtime decrease in the MMSE across the follow-up: those with ≥2 points decrease were considered decliners; the others were considered non-decliners. The positive status of amyloid in plasma was measured as described previously (17).

#### Characteristics of the participants

Outcomes were assessed at the visit. Blood samples were collected at the initial visit and then annually for 5 years. Overall cognitive performance was assessed using: a composite cognitive score (CCS) (18) based on 4 tests (the 10 orientation items of the MMSE, the Digit Symbol Substitution Test, free and total recall of the Free and Cued Selective Reminding Test, and the Category Naming Test); and the MMSE score (19). CCS was computed as the mean *z*-score of the 4 domains, calculated using the baseline mean and *SD* values of the corresponding test. The physical capacities were evaluated by the usual pace gait speed (GS) test, the Short Physical Performance Battery, the 5-repetition sit-to-stand test (5-STS) (20), and the maximal handgrip strength (HS) (measured in kg by a handled dynamometer [Jamar, Bolingbrook, IL]).

#### Measurement of cognitive decline

The MMSE score is a global cognitive scale used worldwide. It is widely used in the literature to describe cognitive decline (21). The cognitive composite score referenced in this article is tailored to the MAPT study and lacks a defined threshold for categorizing our population into 2 groups based on this score. Consequently, in this study, the composite score is exclusively employed to characterize the baseline study population (22). The cognitive composite score serves solely for characterizing the baseline sample and is not utilized for delineating the “no decline” and “decline” subgroups. For this purpose, we used the difference in the MMSE global score assessed longitudinally.

### Metabolomic profiling

#### Extraction of plasma samples

A total of 100 µl of plasma samples were homogenized in 1 ml of methanol. The homogenates were transferred to glass tubes with 900 µl of methanol and 2 ml of dichloromethane was added. The samples were vortexed and centrifuged (1 500*g*, 5 minutes, 4°C). Supernatants were collected and 600 µl of 0.9% NaCl solution was added. Samples were vortexed, and centrifuged (1 500*g*, 5 minutes, 4°C), and aqueous and organic phases were collected into 2 test tubes.

#### Sample preparation of aqueous extracts for NMR analyses

Aqueous phases were dried using the SpeedVac facility. Dried extracts were reconstituted in 200 µl of phosphate buffer (0.2M, pH 7.0) prepared in deuterium oxide (D_2_O) and containing 1 mM of sodium trimethylsilylpropionate (TSP), centrifuged (15 minutes, 2 870*g*, 4°C) and transferred into 3 mm NMR tubes.

#### NMR analyses

^1^H NMR spectra were obtained at 300 K on a Bruker Avance III HD 600 MHz NMR spectrometer (Bruker Biospin, Rheinstetten, Germany), operating at 600.13 MHz for ^1^H resonance frequency using an inverse detection 5 mm ^1^H-^13^C-^15^N-31P cryoprobe. “Tuning” and “matching” of the probe, lock, shims tuning, pulse (90°), and gain computation are automatically performed on each sample. The ^1^H NMR spectra were acquired using the 1D NOESY experiment with presaturation for water removal (noesypr1d), with a mixing time of 100 ms. A total of 128 transients were collected into 64 000 data points using a spectral width of 12 ppm, a relaxation delay of 15 seconds, and an acquisition time of 4.55 seconds. Prior to the Fourier transform, an exponential line broadening function of 0.3 Hz was applied to the free induction decays. All NMR spectra were phase- and baseline-corrected and referenced to the chemical shift of TSP (0 ppm) using Topspin (V3.2, Bruker, Biospin, Germany). The ^1^H NMR spectra were then divided into variable size buckets between 8.5 and 0.7 ppm using the AMIX software (v3.9.15, Bruker, Rheinstetten, Germany), and the area under the curve was calculated for each bucket (integration). Variable-sized bucketing means that each bucket may have an individual size. Buckets were defined graphically as a spectral pattern (excluding solvent signals, and noise), and this pattern was used for bucketing. A total of 102 buckets or variables (several variables can correspond to the same metabolite) were defined with this method. Integrations were normalized according to the total intensity. Preprocessed data were then exported into an Excel file. Spectral assignment was based on matching one-dimensional (1D) data to reference spectra in a home-made database, as well as with other databases (https://bmrb.io/; https://www.hmdb.ca; https://peakforest.org/). Assignments were confirmed by 2D NMR experiments: ^1^H-^13^C HSQC (Heteronuclear Single Quantum Correlation); ^1^H-^1^H COSY (Correlation Spectroscopy; ^1^H-^1^H TOCSY [Total Correlation Spectroscopy]) and ^1^H-^13^C HMBC (Heteronuclear Multiple Bond Correlation). Validation of identification was based on the nomenclature of the metabolomics standards initiative (23). A total of 30 compounds were identified (Level 1), with the same proton and carbon-13 chemical shifts of reference compounds analyzed in the same conditions.

#### Sample preparation for lipidomic analyses

A total of 20% of the organic phase were dried off in the presence of a mixture of internal standards (11:0 LPC (2.5mg/ml), 13:0 LPE (2mg/ml), 12:0 PG (2mg/ml), 13:0 PC((5mg/ml), 12:0 PE (5.5mg/ml), d18:1/12:0 Cer (2mg/ml), d18:1/12:0 lacCer (6mg/ml), d18:1/12:0 GalCer (2.5mg/ml), d18:0/12:0 SM (5mg/ml), TG 17:1:17:0/17:0 d((2.5mg/ml), FA 17:0 (5mg/ml), CE 17:0 (1mg/ml), DG 12:0-12:0 (5mg/ml), PI 15:0-18:1-d7 (1.5mg/ml), PS 12:0-12:0 (3mg/ml)) and dissolved in 100 µl of MeOH:Isoprpanol:H2O (v/v/; 65:35:5).

#### Untargeted lipidomic profiling by Supercritical Fluid chromatography coupled with high-resolution mass spectrometry (SFC-HRMS)

The lipid extract was profiled using supercritical fluid chromatography. One µL of the extract was injected on the Ultra-Performance Convergence Chromatography (UPC2) system coupled online to an Xevo G2-XS time of flight (Qtof; Waters, Milford, MA) equipped with electrospray ionization (ESI). The analysis was performed in both ionization modes (positive and negative) in 2 separate runs on an ACQUITY UPC2 Torus diethylamine column (100 × 3.0 mm inner diameter (i.d.), particle size: sub-1.7µm, Waters) at 40°C. Mobile phases with a flow rate of 1.2 mL/min were constituted by SCCO_2_ for the A phase and MeOH:H_2_O (98:2; v/v) with 20 mM of ammonium acetate for the B phase (modifier). The gradient program was as follows: initial conditions were 1% of B solvent; from 0.5 to 6 minutes it was increased to 40% then from 6 to 6.10 minutes to 65%. The solvent B was maintained to 65% during 3 minutes then the gradient went back to initial conditions in 3 minutes with an active back pressure regulator (ABPR), 1 500 pounds per square inch (psi). From 6 to 9 minutes, the flow rate was decreased to 1.1 mL/minutes. The make-up solvent was MeOH:H_2_O (95:5; v/v) at 0.1 mL/minutes during all run. The source parameters of the mass detector were set as follows: for positive and negative analysis source temperature was 150°C, capillary voltage was at −2.6 kV in negative mode and 3 kV in positive mode, desolvation gas flow rate was 1 000 L/hour, cone gas flow rate was set at 50 L/hour, and the desolvation temperature was 550°C. The analyses were performed in MS full scan in centroid mode from 50 to 1 500 Da with dynamic range extended (DRE) activated. MS/MS experiments were performed in positive and negative ion modes on the same instrument, using a ramp of collision energy ranging from 10 to 50 eV. The isolation width was set at m/z 5. MS/MS mass spectra were inspected manually to confirm annotations. The method allows the separation and profiling of 18 subfamilies of lipids: sterol and sterol ester, diacylglycerid, triacylglycerid, ceramides, phosphatidylcholine (PC), phosphatidylserine, phosphatidylethanolamine (PE), phosphatidylinositol, phosphatidylglycerol, LysoPC, LysoPE, cardiolipin, sphingomyelin, monohexosylceramide, dihexosylceramides, free fatty acids and acyl-carnitine. The lipidomics data were processed with a suspect screening approach, through the interrogation of an in-house database, using MS-DIAL (24) which allows the relative quantification of each species of subclasses families using internal standards (one standard per sub-class of lipids) (25).

### Statistical Analysis

#### Clinical data

Descriptive statistics were provided using mean (*SD*) and absolute values and percentages, as appropriate. The Mann–Whitney U test and Fisher’s exact test were performed as appropriate to examine the differences between decliners in cognitive function and nondecliners.

#### Statistical metabolomic analysis

Multivariate analyses were used to separate patients according to decline status from metabolome or lipidome profiles. First, Principal Components Analysis (PCA) was performed to reveal intrinsic clusters (eg, Sex) and detect eventual outliers. Covariates that differed between cognitive decliners and non-decliners at *p* < .05 were assessed using PCA. Partial least squares–discriminant analysis (PLS-DA) was then used to model the relationship between decline status and spectral data. Data were Pareto-scaled. The R2Y parameter represents the explained variance. Sevenfold cross-validation was used to determine the number of latent variables to include in the PLS-DA model and to estimate the predictive ability (or predicted variance, Q2 parameter) of the fitted model. PLS-DA models with a Q2 value higher than 0.4 were considered valid (26). SIMCA-P software (V14, Umetrics AB, Umea, Sweden) was used to perform the multivariate analyses, and R (https://www.r-project.org/) for univariate testing. The 2 analytical methods used to profile plasma metabolites provide complementary information, since ^1^H NMR is used to profile polar metabolites in aqueous extracts while SFC-HRMS allows to profiling of lipids in organic extracts. Statistical integration of both data sets, that is, simultaneous analysis of NMR and MS data sets, can be very beneficial to increase information about the decline status and so to get more predictive models of decline. We used DIABLO (27) for this purpose. This supervised multivariate method generalizes sparse Canonical Correlation Analysis (CCA) to classification. CCA is used to assess the correlation between block variables, that is, the NMR and MS blocks in this study. The sparse version of CCA performs variable selection. This selection allows the discarding of noisy variables and redundancy within and between the data sets: only predictive variables of the decline status are selected in the final model. DIABLO works in 2 steps: in the first step, the optimal number of latent components is chosen. Then, the optimal number of variables to select in each data set is fixed. These optimal numbers are chosen to minimize the balanced error rate (BER) defined as the average of the errors made on each class (Decliners classified as non-Decliners and non-Decliners classified as Decliners). BER was computed using 4-fold cross-validation. We applied a bootstrap resampling strategy to assess the stability of the optimal numbers of latent components and variables to select. For each bootstrap sample, the optimal number of latent components to include in the model was first fixed (models with 1–5 components were tested). Once the number of latent components was fixed, the number of variables to select in each data set was optimized using a grid of values including 1–9 (step = 1), 10–100 (step = 5) variables for the metabolomic data set and 1–9 (step = 1), 10–290 (step = 5) variables for the lipidomic data set.

The final model (on the entire sample) was fitted based on the bootstrap results. Model performance was evaluated using Aurea under Curve (AUC) values. The mixOmics R package was used for the DIABLO method (28).

## Results

### Characterization of the Samples From MAPT Study Participants

Twenty participants (*n* = 8 decliners in cognitive function; *n* = 12 nondecliners) were included in the placebo group of the MAPT study (Table 1). Decliners and non-decliners did not have statistically significant baseline differences in sociodemographic factors, clinical measures, such as comorbidities, and functional measures that included GS, SPPB (Short Physical Performance Battery), mood, or cognitive function. As shown in Table 1, the mean follow-up duration was 4.7 years for nondecliners and 3.6 years for decliners. However, this difference did not reach statistical significance (*p* value = .094), leading us to exclude follow-up length as a covariate in our model.

**Table 1.** Baseline Characteristics of Decliners (*n* = 8) and Non-decliners (*n* = 12)

	*N*	Overall (*N* = 20)	Evolution of MMSE		
			No decline (*N* = 12)	Decline (*N* = 8)	*p* Value
Age (y), mean (*SD*)	20	77.0 (5.4)	78.3 (5.3)	74.9 (5.0)	.153
Female, *n* (%)	20	10 (50%)	6 (50%)	4 (50%)	1.000
Education, *n* (%)					
No diploma or primary certificate	19	5 (26%)	3 (27%)	2 (25%)	.921
Secondary education		6 (32%)	4 (36%)	2 (25%)	
High school diploma		3 (16%)	1 (9%)	2 (25%)	
University level		5 (26%)	3 (27%)	2 (25%)	
Comorbidities, *n* (%)					
Asthma/COPD	20	1 (5%)	0 (0)	1 (13%)	.400
Diabetes		3 (15%)	2 (17%)	1 (13%)	1.000
Hypertension		10 (50%)	5 (42%)	5 (63%)	.650
Hypercholesterolemia		10 (50%)	5 (42%)	5 (63%)	.650
Ischemic heart disease		3 (15%)	1 (8%)	2 (25%)	.537
Stroke		1 (5%)	0 (0)	1 (13%)	.400
Heart failure		0 (0)	0 (0)	0 (0)	—
Active cancer		0 (0)	0 (0)	0 (0)	—
BMI (kg/m^2^), mean (*SD*)	20	25.7 (2.6)	26.1 (2.7)	25.1 (2.5)	.375
GDS score (0–15), mean (*SD*)	20	3.1 (2.3)	3.3 (2.9)	2.9 (1.2)	.938
MMSE score (0–30), mean (*SD*)	20	27.0 (1.6)	26.9 (1.5)	27.1 (1.8)	.665
FCSRT score, mean (*SD*)					
Free recall (0–48)	20	26.8 (6.4)	28.1 (4.6)	24.9 (8.4)	.438
Total recall (0–48)	20	45.8 (3.9)	46.8 (1.2)	44.1 (5.9)	.219
Delayed free recall (0–16)	20	9.8 (2.6)	10.3 (2.0)	9.1 (3.3)	.350
Delayed total recall (0–16)	20	15.6 (1.1)	15.8 (0.4)	15.1 (1.7)	.284
TMT Part A, mean (*SD*)	20	48.7 (12.4)	47.0 (13.4)	51.3 (11.2)	.462
TMT Part B, mean (*SD*)	20	122.5 (36.4)	121.3 (33.3)	124.3 (43.1)	.908
WAIS-R, mean (*SD*)	20	33.1 (5.7)	33.2 (6.4)	32.9 (4.9)	.938
COWAT score, mean (*SD*)	20	18.7 (5.3)	18.5 (6.5)	19.0 (3.3)	.816
CNT score, mean (*SD*)	20	24.5 (6.5)	26.1 (6.6)	22.1 (5.8)	.245
CDR score, *n* (%)					
Score 0	20	8 (40%)	4 (33%)	4 (50%)	.648
Score 0.5		12 (60%)	8 (67%)	4 (50%)	
SPPB score (0–12), mean (*SD*)	19	10.2 (1.6)	9.7 (1.8)	10.8 (1.0)	.234
Gait speed (m/s), mean (*SD*)	19	1.0 (0.3)	1.0 (0.3)	1.0 (0.3)	.772
Chair stand time (sec), mean (*SD*)	19	10.9 (3.4)	11.8 (3.6)	9.8 (3.0)	.173
Balance test (0–4), mean (*SD*)	20	3.4 (0.9)	3.3 (1.1)	3.6 (0.7)	.440
Handgrip strength (kg), mean (*SD*)	18	28.8 (11.6)	29.4 (10.1)	27.5 (15.2)	.606
Fried frailty phenotype, *n* (%)					
Robust (0/5)	17	8 (47%)	6 (55%)	2 (33%)	.620
Pre-frail (1–2/5)		9 (53%)	5 (45%)	4 (67%)	
Frail (≥3/5)		0 (0)	0 (0)	0 (0)	
APOE status					
ε2	17	0 (0)	0 (0)	0 (0)	.762
ε2/ε3		1 (6%)	1 (10%)	0 (0)	
ε2/ε4		0 (0)	0 (0)	0 (0)	
ε3		11 (65%)	7 (70%)	4 (57%)	
ε3/ε4		5 (29%)	2 (20%)	3 (43%)	
ε4		0 (0)	0 (0)	0 (0)	
APOE ε4 carrier (ε2/ε4, ε3/ε4, ε4), *n* (%)	17	5 (29%)	2 (20%)	3 (43%)	.593
Cortical SUVR, mean (*SD*)	12	1.34 (0.16)	1.27 (0.17)	1.41 (0.14)	.298
SUVR positive (≥1.17), *n* (%)	12	10 (83%)	4 (67%)	6 (100%)	.455
Plasma amyloid-beta, mean (*SD*)	20	0.10 (0.01)	0.10 (0.01)	0.10 (0.01)	.557
Follow-up length (y)	20	4.3 (1.3)	4.7 (0.8)	3.6 (1.6)	.094

*Notes*: APOE = Apolipoprotein E; BMI = body mass index; CDR = Clinical Dementia Rating scale; CNT = Category Naming Test; COPD = chronic obstructive pulmonary disease; COWAT = Controlled Oral Word Association Test; FCSRT = Free and Cued Selective Reminding Test; GDS = Geriatric Depression Scale; MAPT = Multidomain Alzheimer Preventive Trial; MMSE = Mini Mental State Examination; *SD* = standard deviation; SPPB = Short Physical Performance Battery; SUVR = standard uptake value ratio; TMT = Trail Making Test; WAIS-R = Wechsler Adult Intelligence Scale-Revised.

*p* Values determined using Fisher’s exact test for categorical variables or using Mann–Whitney U test for continuous variables.

### Aqueous Metabolomics or Lipidomic Profiling Alone Cannot Predict Future Cognitive Decline

We used dual technology for the same blood sample, which allows us to cover a very large number of metabolites and assess the different lipid species. ^1^H-NMR provides relative quantification of metabolites based on the intensity of the spectral peaks while SFC-HRMS offers large metabolite coverage, sensitivity, and selectivity. So, to provide complementary information about aqueous and lipid metabolites and to broaden metabolite coverage, we performed unbiased plasma metabolomic profiling of aqueous and lipid metabolites, which allowed the identification of 30 polar metabolites and 290 lipid species. No covariate exhibits a significant difference between groups at *p* < .05; hence, none of the covariates was taken into consideration. Individual PCA was first performed on each data set to describe the global variability and information on the decline status contained in the profiles. The 2D score plots showed that participants could not be separated according to cognitive decline status, indicating that the main variability is independent of cognitive status (Supplementary Figure 1). The supervised PLS-DA method was used to model the relationship between the decline status and spectral profiles. No significant model PLS-DA could fit data, meaning that neither the aqueous nor the lipidomic data set contained any predictive signature of the decline status.

### Integration of the Metabolomic Data sets Allows Prediction of Future Cognitive Decline

**Figure 1.**
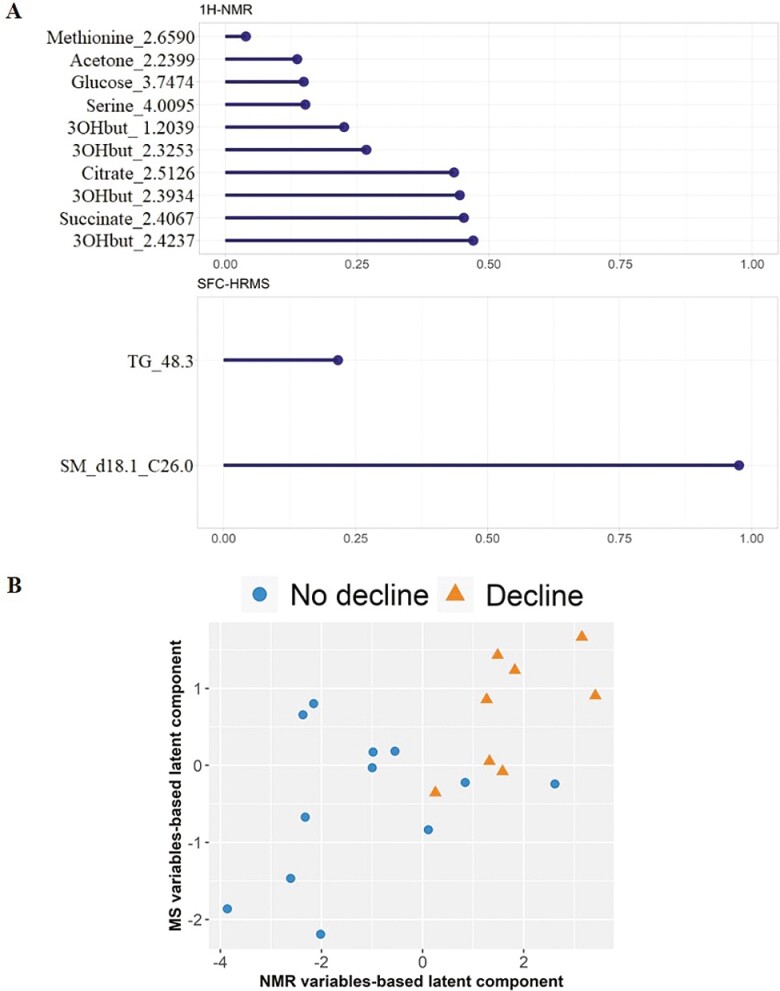
A totsl of 9 metabolites across the NMR and SFC-HRMS data sets together predict cognitive decline. (**A**) Variable importance plots for the cognitive decline metabolome and lipidome biomarker panel. The most important variables (according to the absolute value of their coefficients) are ordered. Absolute loading plot for the 1H-NMR (top) and SFC-MS (bottom) variables selected by multiblock sPLS-DA (DIABLO) performed on the MAPT study (non-decliner and decliner patients). The most important variables (according to the absolute value of their coefficients) are ordered from bottom to top. (**B**) Score plot from multiblock sPLS-DA. Samples are represented based on the specified component (here 1 latent component). Samples are colored by decline status. 3OHbut: 3-hydroxybutyrate; TG: Triglyceride; SM: Sphingomyelin.

The 2 omics data sets contained complementary information and can provide a more comprehensive and detailed understanding of the metabolome, therefore we next tested whether a predictive signature of the decline status could be defined by statistically integrating both data sets. We applied the multi-omics integrative Data Integration Analysis for Biomarker discovery using the Latent cOmponents (DIABLO) method on Pareto-scaled data. This method seeks common information across different data types through the selection of a subset of molecular features, while discriminating between multiple phenotypic groups (27). The bootstrap resampling method was used to assess the robustness of the number of latent components and the number of variables to select per block. These optimal numbers were chosen to minimize the 4-fold cross-validation-based BER. Models with 1 latent component including 1–20 NMR variables and 1–2 MS variables (Supplementary Figure 2) were tested to select the optimal number of variables to select in the NMR and in the MS data sets. We observed that for most of the bootstrap samples, less than 20 variables in NMR block (53%) were selected and 2 variables in the lipidomic block (40%). Sphingomyelin d18.1_C26.0 and triglyceride 48.3 were the most frequently selected in the models adjusted on bootstrap samples (60%, Supplementary Figure 3). This means that these 2 lipids (sphingomyelin d18.1_C26.0 and triglyceride 48.3) are the most predictive of the decline status. Ultimately, 12 variables, including 10 from the NMR data set and 2 from the MS data set, together represented the best possible combination to predict decline (Figure 1A) and enabled discrimination of patients according to the decline status (Figure 1B). These variables corresponded to 7 aqueous metabolites and 2 lipids from 320 initial molecules (Table 2). Among the discriminating variables, 4 different buckets/variables were obtained for 3 hydroxybutyrate. These 9 metabolites were the most frequently selected in the models adjusted on bootstrap samples (≥60%, Supplementary Figure 3).

**Table 2.** Modulation of endogenous metabolites quantified in (**A**) NMR spectra and (**B**) endogenous lipids quantified by SFC-HRMS profiling of plasma samples and selected by multiblock sPLS-DA. Fold change (FC) corresponds to the ratio of the mean NMR areas or MS intensities (Decline/No decline). SM = Sphingomyelin; TG = Triglycerides

A				
Metabolite	Chemical shift (ppm)	FC	Superpathway	Subpathway
3-Hydroxybutyrate	1.20 ; 2.32 ; 2.39 and 2.42	0.72	Lipids	Ketone bodies
Citrate	2.51	0.81	Energy	TCA cycle
Succinate	2.41	0.79	Energy	Succinate pathway
Acetone	2.24	0.85		Ketone bodies
Methionine	2.66	0.92	Amino acid	Methionin pathway
Glucose	3.75	1.09	Carbohydrate	Glycolysis, gluconeogenesis, metabolism
Serine	4.01	1.11	Amino acid	Methionin pathway

B				
Lipids	FC	Superpathway	Subpathway	
SM (d18:1/C26:0)	1.535	Lipids	Sphingolipid metabolism	
TG (C48:3)	1.536	Lipids		

### The Prediction Model Performs Well in Discriminating No Decline Versus Decline Groups

**Figure 2.**
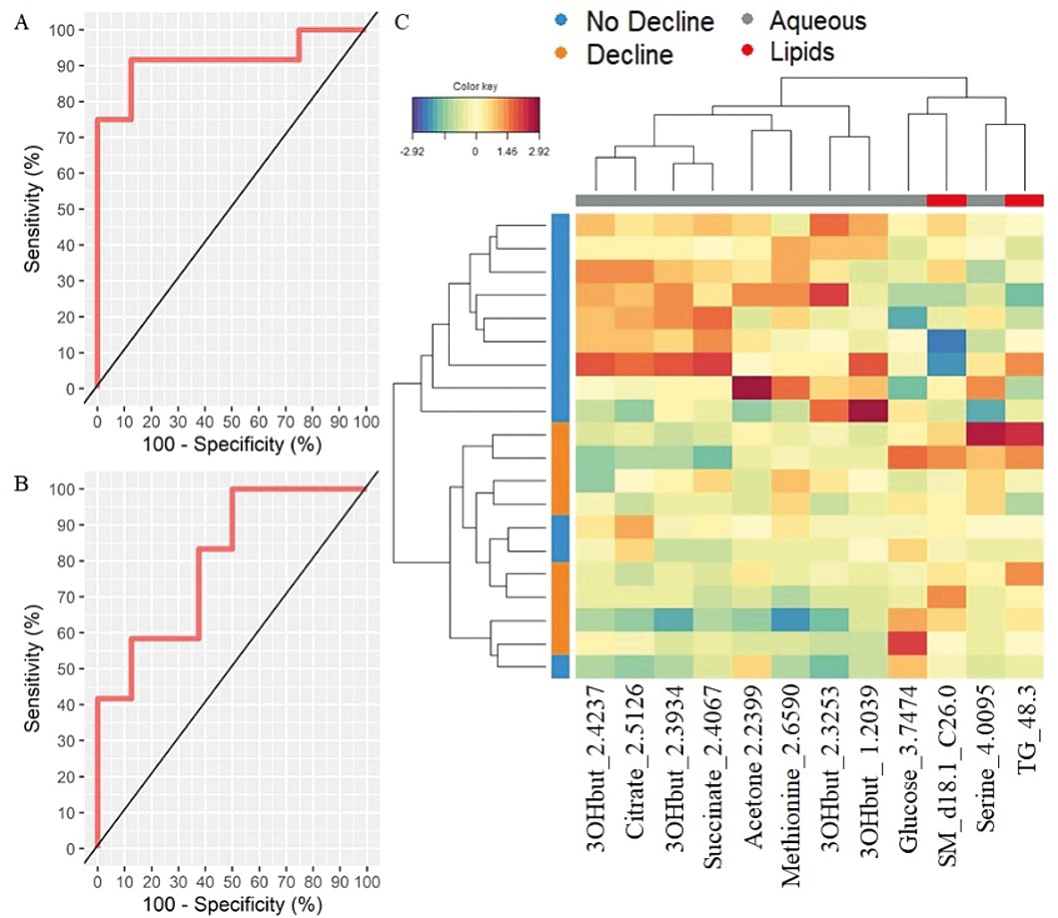
The sPLS-DA model accurately predicts groups without decline from groups with decline. Performance of the multiblock sPLS-DA model and Clustered Image Map (CIM) of metabo-clinical signatures. (**A**) ROC based on multiblock sPLS-DA model for the NMR data set (AUC = 0.9167; *p* value = .002028). (**B**) ROC based on multiblock sPLS-DA model for the Lipidomic data set (AUC = 0.8021; *p* value = . 02526). (**C**) Clustered Image Map for the variables selected by multiblock sPLS-DA on component 1. The CIM represents samples in rows (indicated by their Decline status on the left hand side of the plot) and selected features in columns (indicated by their data type at the top of the plot): 2.4237: 3-hydroxybutyrate; 2.5126: Citrate; 2.3934: 3-hydroxybutyrate; 2.4067: Succinate; 2.2399: Acetone; 2.6590: Methionine; 2.3253: 3-hydroxybutyrate; 1.2039: 3-hydroxybutyrate; 3.7474: Glucose; d18.1_C26.0: Sphingomyelin; 4.0095: Serine; 48.3: Triglyceride.

We then used receiver operating characteristic (ROC) curves to evaluate the performance of the prediction model. The results showed very good predictive ability with an AUC of 0.9167 with a *p* value = .002028 for NMR (Figure 2A) and an AUC = 0.8021 with a *p* value = .02526 for the lipidomic data sets (Figure 2B). We used a clustered image map to discriminate between the No Decline and Decline groups and to obtain the metabolic signature of each sample. Figure 2C shows discrimination between the 2 groups. These results indicate that concentrations of 3-hydroxybutyrate, citrate, succinate, acetone, and methionine are lower in the Decline group whereas those of glucose, serine, sphingomyelin d18.1_C26.0, and triglyceride 48.3 are higher.

## Discussion

This exploratory study identifies a plasma molecular signature that predicts cognitive decline in amyloid-positive individuals. Using untargeted metabolomics with the combination of 2 complementary NMR and SFC-HRMS methods and a mixOmics framework for the integration of multiple data sets (DIABLO), we could clearly identify a minimal signature of 9 metabolites out of 320 that distinguished people who declined at least 2 points in the MMSE over 4 years from those who did not. One of the reasons why the integration of the 2 data sets and the use of DIABLO are possibly more effective may stem from the fact that, through the integration of the 2 data sets, we enhance the coverage of the metabolome, thereby acquiring more information about the decline effect. Additionally, variable selection is implemented in the DIABLO method, enabling the exclusion of noisy and “confounded” variables (those unaffected by cognitive decline and therefore not predictive of decline). This differs from classical multivariate methods like PCA and PLS-DA, where such variable exclusion is not explicitly carried out. To prevent the occurrence of overfitting resulting from our relatively small sample size, we implemented a combination of bootstrap resampling and cross-validation in our study. This approach was employed to finely tune the hyperparameters of the DIABLO model, which involved determining the number of canonical components and selecting the 1H-NMR buckets and MS lipids to be incorporated into these components. The final model’s performance (AUC) was evaluated using a 2-fold cross-validation, repeated 10 times. This strategy allowed us to develop a model with a slightly biased predictive performance, indicated by Bias(AUC1H-NMR) = −0.004 and Bias(AUCSFC-MS) = −0.04.

Cognitive function is affected by both hereditary and environmental parameters (29,30). Given the diversity of phenotypes associated with cognitive decline, no single biomarker can fully reflect this complex pathophysiological process in amyloid-positive participants subjects. Mild cognitive impairment (MCI) and AD are both associated with changes in metabolic biomarkers. The metabolome reflects interactions between genetics, epigenetics, and the environment, and understanding the relationship between metabolic profiles and cognitive decline can lead to improved biomarkers for cognitive decline or AD before the onset of overt clinical signs.

A key result of this study is the use of an untargeted metabolomics approach to predict future decline. To date, targeted metabolomics approaches have been used to describe cognitive decline, however, unbiased metabolomic approaches are less frequently applied. Several targeted metabolic studies have identified plasma metabolic biomarkers associated with current MCI or Alzheimer’s disease status (13,31–34). Previous work has also identified a set of 10 lipids that predicted MCI, however, these results could not be subsequently replicated (35–37). An elegant recent study by Buergel T. et al. used NMR to analyze blood samples from individuals in the UK biobank cohort over a 12-year period. They identified metabolites and lipids that can predict the trajectory of individuals toward dementia (6), including dysregulation of major lipid families such as sphingomyelins and triglycerides. However, the addition of global lipidomics in our study enabled us to precisely identify which type of sphingomyelin and triglycerides were associated with the prediction of cognitive decline.

Our integrative multimodal metabolomic approach enabled us to precisely identify sphingomyelin d18:1/C26:0 and triglyceride C48:3. Likewise, methionine was 1 of the top 10 selected variables (approximately 70%, Supplementary Figure 3) in the NMR bootstrapped samples by the DIABLO method. For the individual statistical analysis of each data block, no valid PLS-DA model could be fit, meaning that the information contained in the NMR or MS data alone was not sufficient to build a valid and robust model linking the decline and the spectral data. Combining metabolomic and lipidomic data sets with an innovative statistical integration method enabled us to increase information on decline status, and then the ability to predict Decline status. The combined polar and lipids panel made accurate predictions on average between 3.3 and 4.8 years before the onset of cognitive decline. Thus, combining distinct types of untargeted metabolomics data with longitudinal clinical information has been an important step toward the major clinical challenge of understanding the metabolic changes that precede and can predict cognitive decline.

**Figure 3.**
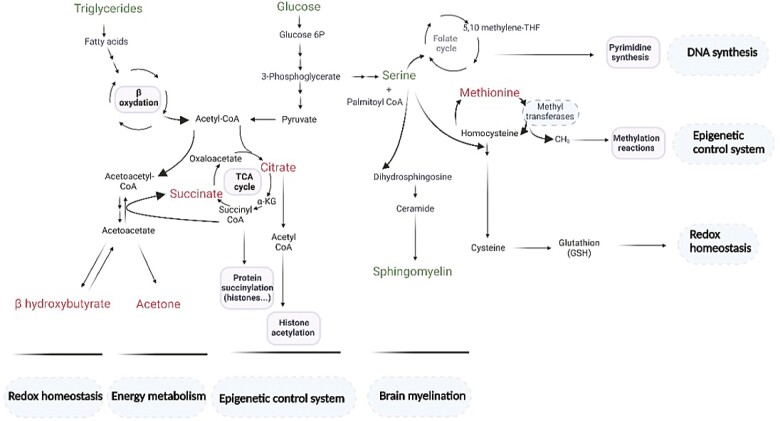
Integrated representation of the metabolic and molecular processes associated with the 9 metabolites composing the early predictive signature of cognitive decline. In green (metabolites showing decline-related increase: glucose, serine, triglycerides, and sphingomyelin) and in red (metabolites showing decline-related decrease: β-hydroxybutyrate, acetone, citrate, succinate, and methionine). Made with Biorender.com.

Some of the metabolites identified in the present study, including glucose, methionine, β-hydroxybutyrate, sphingomyelins, and triglycerides have already been reported to be dysregulated in patients showing impaired cognition and in AD (38–42). From a physiological and integrated perspective, the 9 metabolites identified in this work are involved in the maintenance of brain myelination, the epigenome, and redox homeostasis that regulate most of the body’s key biological processes (Figure 3). Indeed, lipids and particularly sphingolipids, including sphingomyelins, are crucial structural components of neural tissues and myelination and significantly affect cognitive function (43). Moreover, numerous studies have shown that the brain epigenome contributes to age-related memory decline, a major risk factor for the development of Alzheimer’s disease (44). Epigenetic mechanisms such as histone modifications, acetylation, and methylation, play a role in synaptic plasticity and memory formation (45). Finally, ketone bodies, such as β-hydroxybutyrate, which affects cognitive function as a signaling molecule are not only metabolic intermediates, as they regulate a wide range of physiological processes at the cellular and systemic levels. In particular, β-hydroxybutyrate functions as a stress response molecule and orchestrates an antioxidant defense program to maintain redox homeostasis in response to environmental and metabolic challenges. More precisely, β-hydroxybutyrate is a direct antioxidant for hydroxyl radicals and suppresses mitochondrial ROS in stressed neurons (46,47). Based on this metabolic signature, it may be possible to select innovative therapies for patients with AD at risk for cognitive decline. In the future, it will be of great interest to investigate a causative link between metabolism and cognitive decline.

## Limitations

As our study was conducted on 20 individuals, our results must be interpreted with some limitations. Given the exploratory nature of this study, no formal sample size calculation was performed; future confirmatory investigations may take advantage of our results to calculate their study sample. This original metabolomics strategy on plasma will have to be reproduced in other cohorts with a larger population to validate the set of metabolites and the prediction models. Biomarker validation in different populations, such as in the INSPIRE cohort (48), is a major challenge. Understanding the underlying mechanisms of how these metabolites influence cognitive decline will require further investigation.

## Conclusion

This longitudinal study uniquely provides robust predictive models of cognitive decline using untargeted metabolomics by combining both NMR and mass spectrometry and focusing on amyloid-positive individuals. We identified a minimum signature of polar and lipids metabolite that could both predict cognitive decline and provide information on the putative mechanisms leading to AD pathophysiology. Furthermore, its longitudinal nature with a relatively long follow-up and several time points of data collection allows us to learn about the trajectories of different outcome measures of cognitive function and clearly identify a specific metabolic signature as a novel and predictive biomarker of cognitive decline.

## Supplementary Material

glae077_suppl_Supplementary_Figures_S1-S3
