# Supplementary material for: Integrative Multimodal Metabolomics to Early Predict Cognitive Decline Among Amyloid Positive Community-Dwelling Older Adults
Source: J Gerontol A Biol Sci Med Sci. 2024 Mar 7;79(5):glae077. doi: 10.1093/gerona/glae077 (PMC11000317; doi:10.1093/gerona/glae077)
Supplement: glae077_suppl_Supplementary_Figures_S1-S3 [file glae077_suppl_supplementary_figures_s1-s3.zip › SUPPLEMENTARY FIGURES/SUPPLEMENTARY FIGURES.docx]

**SUPPLEMENTARY FIGURES**

**Figure S1:** **Unbiased plasma metabolomic profiling of either aqueous or lipid metabolites is not sufficient to predict cognitive decline.** Two-dimensional PCA score plot of plasma samples (control, n = 12; decline, n = 8). A) Integrated ^1^H NMR spectra of aqueous extracts (2 principal components; R2X = 55.6%). B) SFC-HRMS spectra of lipidic extracts (2 principal components; R2X = 56.7%).

**Figure S2**: Number of selected variables by data set. One hundred bootstrap samples. **A.** 1H-NMR; **B.** SFC-HRMS.

**Figure S3**: Frequency of selection of variables from each dataset in multiblock sPLS-DA models fitted to bootstrap samples. One hundred bootstrap samples. **A.** 1H-NMR; **B.** SFC-HRMS.
