# Supplementary figures and images for: Integrative Multimodal Metabolomics to Early Predict Cognitive Decline Among Amyloid Positive Community-Dwelling Older Adults
Source: J Gerontol A Biol Sci Med Sci. 2024 Mar 7;79(5):glae077. doi: 10.1093/gerona/glae077 (PMC11000317; doi:10.1093/gerona/glae077)

Figure S1

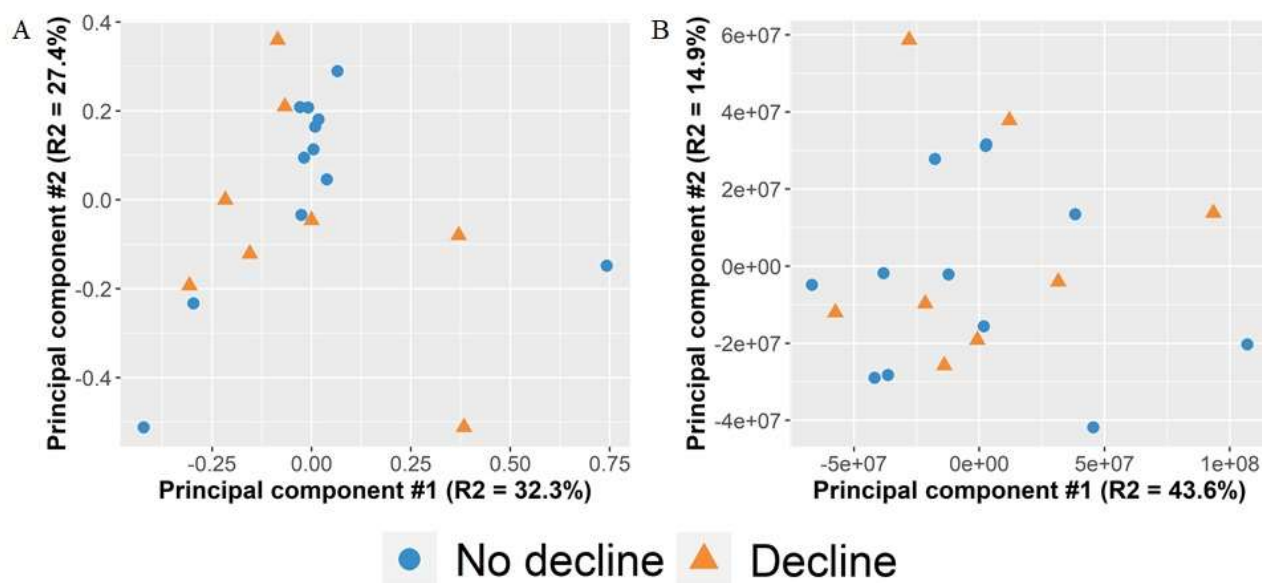

**Figure S2**

**A**

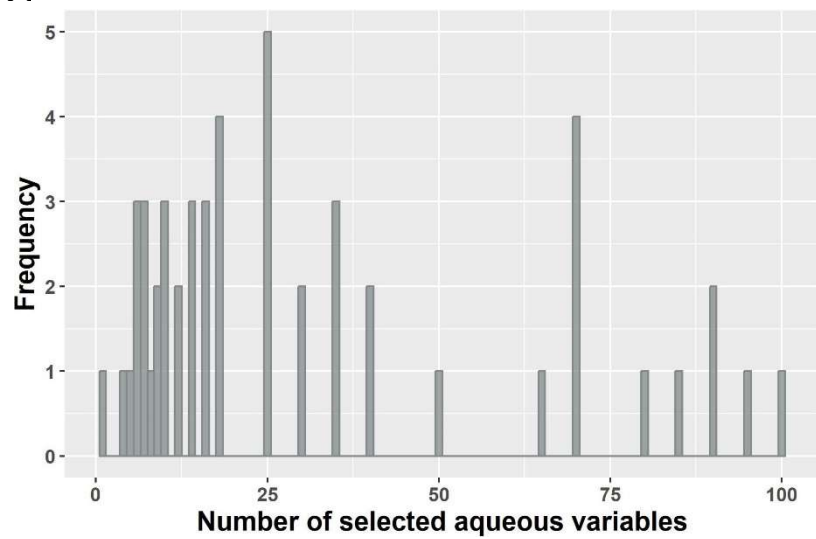

**B**

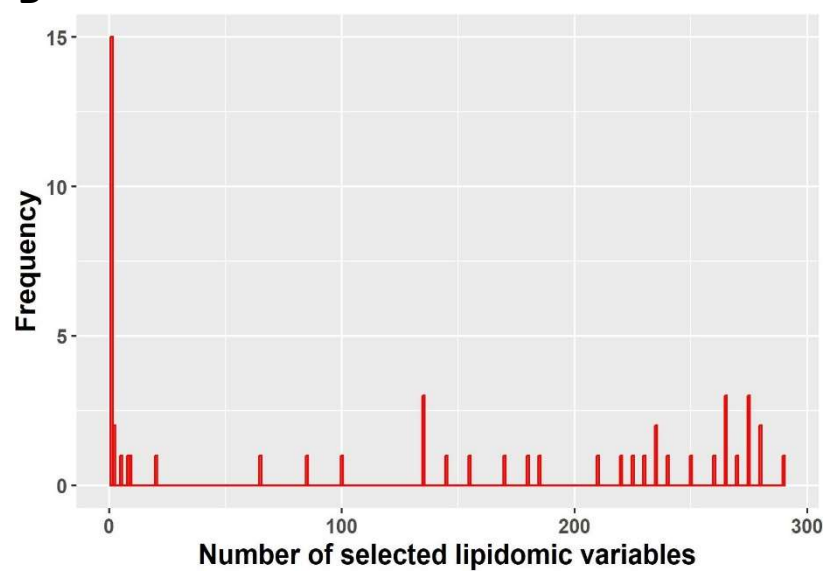

### Figure S3

**A**

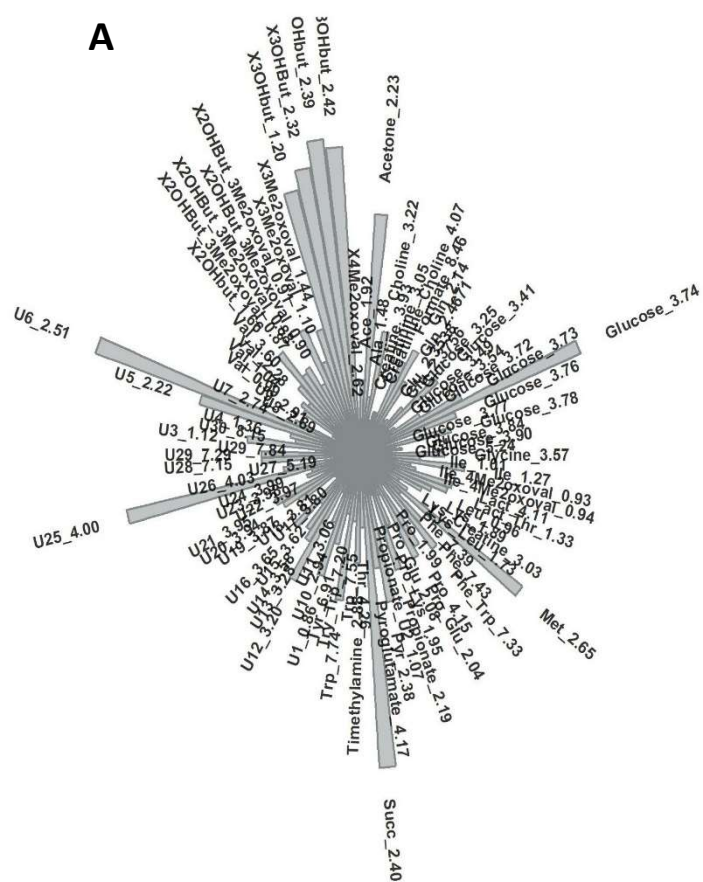

**B**

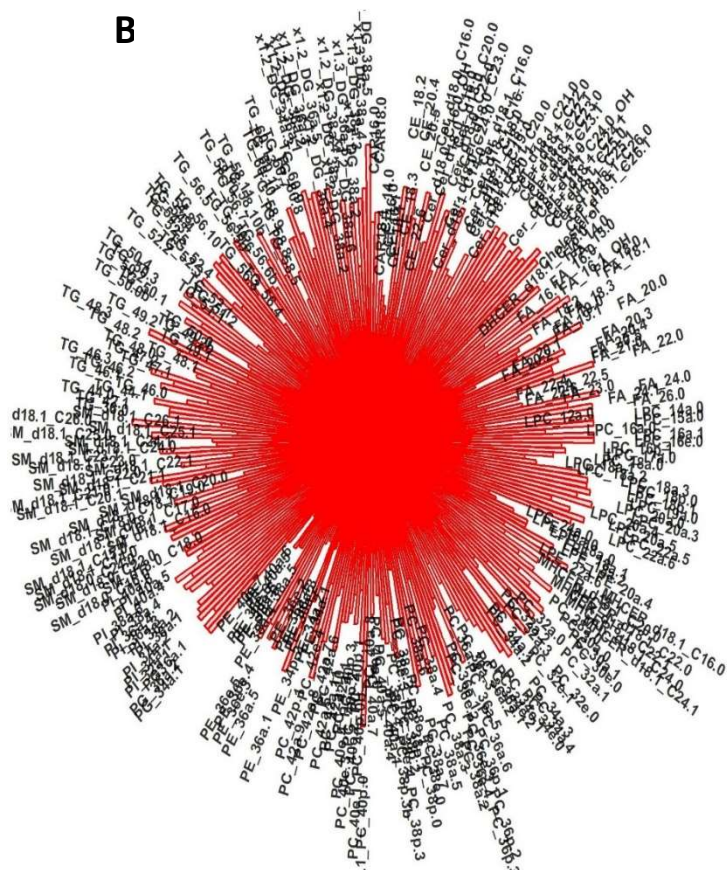

Supplement: glae077_suppl_Supplementary_Figures_S1-S3 [file glae077_suppl_supplementary_figures_s1-s3.zip › SUPPLEMENTARY FIGURES/Tremblay-Franco M_Canlet C et al., Supplementary.pdf]
